# Label-Free Biochemical Characterization and Subtype Classification of Human Breast Pathologies Using Fingerprint Raman Spectroscopy and Machine Learning

**DOI:** 10.3390/bioengineering13101203

**Published:** 2026-10-16

**Authors:** Nguyen Van Hung, Mai Huu Thuan, Ngo Duc Quan

**Affiliations:** 1 Department of Radiation Physics, K Hospital (Tan Trieu Campus), Hanoi 100000, Vietnam; hungvlktk50@gmail.com; 2 Faculty of Engineering Physics, Hanoi University of Science and Technology, No. 1 Dai Co Viet, Hanoi 100000, Vietnam; thuan.maihuu@hust.edu.vn

**Keywords:** Raman spectroscopy, breast pathology, biochemical characterization, machine learning, support vector machine, leave-one-patient-out validation

## Abstract

Breast cancer remains a major cause of cancer-related mortality, highlighting the need for improved molecular characterization of breast pathologies. Raman spectroscopy (RS) provides a label-free, non-destructive approach for probing tissue biochemical composition. This study investigated Raman spectral alterations across five human breast histopathological categories and explored their potential for machine learning-assisted classification. Formalin-fixed, paraffin-embedded (FFPE) tissue specimens were obtained from five patients, with one patient representing each category: normal breast tissue, intraductal papilloma, high-grade ductal carcinoma in situ (DCIS), invasive ductal carcinoma (IDC), and invasive lobular carcinoma (ILC). A total of 100 Raman spectra (20 per patient) were acquired using 785 nm excitation over the fingerprint region (500–2000 cm^−1^). Spectral preprocessing involved Savitzky–Golay smoothing, asymmetrically reweighted penalized least-squares (arPLS) baseline correction, and vector normalization. The investigated spectra exhibited reduced relative lipid-associated contributions near 1063, 1300, and 1445 cm^−1^, accompanied by increased relative protein- and nucleic-acid-associated features near 1003, 1420, and 1600–1650 cm^−1^. The phenylalanine-to-lipid intensity ratio (I_1003_/I_1445_) increased from ≤0.2 in normal tissue to ≥1.5 in invasive lesions, suggesting a potential spectroscopic descriptor of biochemical remodeling. Exploratory subtype-associated differences were also observed, including a prominent aromatic C=C-associated feature near 1618 cm^−1^ in IDC and an enhanced carbohydrate-associated contribution near 890 cm^−1^ in ILC. A support vector machine (SVM) classifier was evaluated using patient-independent leave-one-patient-out (LOPO) cross-validation. Across the five held-out patients, the model correctly classified 89 of 100 spectra, achieving an overall spectrum-level accuracy of 89.0%, sensitivity of 90.0%, specificity of 87.5%, and F1-score of 90.8%. Although these findings demonstrate encouraging discrimination within the investigated cohort, the limited number of biologically independent patients and the representation of each histopathological category by a single patient restrict the generalizability of the results. This study provides preliminary proof-of-concept evidence supporting the integration of Raman-derived biochemical features with machine learning for breast pathology characterization, warranting further validation in larger, multi-patient and multicenter cohorts.

## 1. Introduction

Breast cancer remains one of the most common malignancies and a leading cause of cancer-related mortality among women worldwide. According to GLOBOCAN 2022, approximately 2.3 million new breast cancer cases and 670,000 associated deaths were estimated globally in 2022 [1]. In Vietnam, breast cancer is the most frequently diagnosed malignancy among women, with approximately 24,500–25,000 new cases and 10,000 deaths reported annually [1]. Early detection and accurate pathological characterization are essential for improving treatment outcomes and patient management [1,2]. Conventional diagnostic modalities, including mammography, ultrasonography, magnetic resonance imaging (MRI), and histopathological examination, play indispensable roles in breast cancer diagnosis. Nevertheless, these methods have limitations related to diagnostic specificity, procedural invasiveness, resource requirements, and interobserver variability, depending on the modality [1,2,3]. Consequently, complementary analytical techniques capable of providing objective molecular information about breast tissues remain of considerable research interest.

Among emerging biospectroscopic approaches, Raman spectroscopy (RS) has attracted attention because of its ability to probe the intrinsic biochemical composition of biological specimens through molecular vibrational signatures [3,4]. Based on the inelastic scattering of incident photons, Raman spectroscopy produces characteristic spectral bands associated with molecular bonds and biochemical constituents, providing a label-free molecular fingerprint of the investigated tissue [3]. Unlike conventional histological staining, Raman measurements can provide information about biochemical composition without requiring molecular labels. This capability makes Raman spectroscopy a promising complementary technique for investigating biochemical alterations associated with breast pathological states.

Breast malignancies are associated with complex biochemical and metabolic alterations involving proteins, lipids, nucleic acids, and carbohydrates [5]. These alterations may be reflected in Raman spectra through changes in band intensities, positions, and widths [5,6]. Several characteristic Raman bands have been associated with biologically relevant molecular constituents, including the band near 785 cm^−1^, commonly attributed to nucleic-acid-related vibrations; the phenylalanine-associated band near 1002–1003 cm^−1^; the CH_2_ deformation band near 1445 cm^−1^, associated with lipid and protein contributions; and the Amide I region near 1655 cm^−1^, which is sensitive to protein structure [5,7]. Changes in the relative contributions of these spectral features may provide information about biochemical remodeling in pathological tissues. In particular, ratios between selected Raman-band intensities offer potentially interpretable descriptors of relative biochemical composition, although their biological specificity and diagnostic utility require independent validation.

Despite these advantages, Raman spectra obtained from biological tissues are often affected by fluorescence backgrounds, instrumental noise, spectral variability, and intrinsic tissue heterogeneity [8,9,10]. Such factors can obscure weak biochemical signals and complicate direct spectral interpretation. Computational preprocessing and multivariate analytical methods are therefore important for extracting meaningful information from complex Raman datasets. Common processing strategies include spectral smoothing, fluorescence-background correction, normalization, and feature extraction, followed by dimensionality reduction and statistical classification [1,3,8,9,10].

Artificial intelligence (AI) broadly encompasses computational methods designed to perform tasks associated with human intelligence, including pattern recognition, prediction, and decision support. Machine learning (ML), a major subset of AI, enables algorithms to learn statistical relationships from data and apply these relationships to previously unseen observations. In healthcare, AI and ML have been investigated for medical image interpretation, disease detection, risk stratification, electronic health record analysis, and clinical decision support. For example, the study [11] illustrates the application of ML to identifying clinically challenging infections using hospital data. Similarly, recent work [12] demonstrates the potential of causal ML approaches for supporting decisions in intensive care settings. Although these applications involve different data modalities and clinical objectives, they highlight the broader role of data-driven methods in extracting actionable information from complex healthcare datasets. In molecular spectroscopy, ML can likewise assist in identifying discriminative spectral patterns that may be difficult to resolve through conventional univariate analysis.

The integration of Raman spectroscopy with ML has consequently attracted increasing attention in cancer-related research [13,14]. Algorithms such as linear discriminant analysis (LDA), support vector machines (SVMs), random forests (RFs), and k-nearest neighbors (KNNs) can be used to classify tissue-associated spectral patterns, while principal component analysis (PCA) can reduce feature dimensionality and reveal dominant sources of spectral variance [3,5,15]. Deep learning (DL) approaches, including convolutional neural networks (CNNs), have also been investigated for learning complex spectral representations directly from experimental data. For example, a previously reported random-stacked convolutional neural network (RS-CNN) achieved a classification accuracy of 98.63% using a relatively large Raman spectral dataset, while its reported performance decreased to 91.47% when fewer spectra were available [7,16]. Raman spectroscopy combined with chemometric and ML methods has also shown promising performance in selected studies of prostate and gastric cancers [1,3,15]. However, classification results from different studies cannot be directly compared without considering differences in sample size, patient composition, acquisition conditions, and validation design.

Importantly, the scientific value of Raman–ML analysis extends beyond classification accuracy. Interpretable spectral features can help connect computational predictions with underlying biochemical differences. For instance, intensity-based descriptors and selected Raman bands may reflect relative contributions from proteins, lipids, nucleic acids, and other tissue constituents [5,7]. Feature-importance analysis and explainable AI approaches, including SHAP-based methods, have also been investigated to identify spectral regions contributing to model predictions [7]. Nevertheless, a discriminative spectral feature should not automatically be regarded as a validated disease-specific biomarker, particularly when biological sample sizes are limited.

A further methodological challenge concerns the distinction between the number of acquired spectra and the number of biologically independent specimens. Multiple Raman spectra obtained from different locations within the same tissue can characterize spatial heterogeneity, but they are not independent patient observations. Consequently, random spectrum-level partitioning may place measurements from the same patient in both training and testing subsets, potentially producing overly optimistic estimates of model performance. Patient-independent validation strategies, such as leave-one-patient-out (LOPO) cross-validation, offer a more appropriate framework for evaluating generalization to held-out individuals. However, even patient-independent validation remains exploratory when only a small number of patients are available, particularly when histopathological categories are represented by single individuals.

These considerations are especially relevant to formalin-fixed, paraffin-embedded (FFPE) breast tissues, where sample preparation, residual background contributions, biological heterogeneity, and instrumental variation may influence spectral measurements [13]. Although Raman-based approaches have demonstrated potential for distinguishing malignant from non-malignant tissues, further investigation is required to characterize interpretable biochemical differences among normal breast tissue, benign lesions, carcinoma in situ, and invasive carcinomas. In addition, spectral differences between invasive ductal carcinoma (IDC) and invasive lobular carcinoma (ILC) remain of interest because these histopathological subtypes exhibit distinct morphological and biological characteristics. Nevertheless, robust identification of subtype-associated spectroscopic signatures requires data from multiple independent patients within each subtype.

Against this background, the present study investigates Raman spectral characteristics across five representative breast histopathological categories: normal breast tissue, intraductal papilloma, high-grade ductal carcinoma in situ (DCIS), IDC, and ILC. Specifically, we aim to: (i) characterize the Raman spectral profiles of FFPE breast tissues and examine variations in lipid-, protein-, nucleic-acid-, and carbohydrate-associated features; (ii) implement a reproducible preprocessing procedure combining Savitzky–Golay smoothing, asymmetrically reweighted penalized least-squares (arPLS) baseline correction, and vector normalization; (iii) investigate selected Raman-band intensities and biochemical intensity ratios as exploratory spectroscopic descriptors; and (iv) evaluate the discriminative potential of Raman-derived features using supervised machine learning and patient-independent LOPO cross-validation. The study includes 100 Raman spectra acquired from five patients, with one patient representing each histopathological category. Accordingly, its primary contribution is an exploratory, biochemically interpretable Raman–ML framework rather than a clinically validated diagnostic model. The findings are intended to inform future investigations involving larger, histopathologically balanced, and independently validated patient cohorts.

## 2. Materials and Methods

### 2.1. Physical Principles of Raman Spectroscopy

The physical foundation of Raman spectroscopy in oncology resides in inelastic scattering of photons following their interaction with molecular vibrational modes. When incident photons with energy E_0_ = hν_0_ interact with breast tissue, the predominant outcome is elastic Rayleigh scattering. However, a negligible fraction (approximately 10^−7^) undergoes energy exchange—the Raman effect [17]. This interaction produces Stokes and Anti-Stokes shifts; the former is primarily utilized in clinical diagnostics due to its higher intensity at physiological temperatures, as governed by Boltzmann distribution. The Raman shift (Δν), measured in wavenumbers (cm^−1^), is independent of excitation wavelength and acts as a direct probe of vibrational frequencies of specific chemical bonds (e.g., C-H, C=C, P-O_2_). In breast cancer analysis, the Raman spectrum functions as a high-resolution “molecular fingerprint,” capturing the intrinsic biochemical transition from healthy to pathological states [3].

### 2.2. Study Subjects and Specimen Collection

Formalin-fixed, paraffin-embedded (FFPE) breast tissue specimens were obtained from five patients undergoing diagnostic biopsy at K Hospital (Tan Trieu Campus), Hanoi, Vietnam. Each patient contributed one tissue specimen corresponding to one of the five investigated histopathological categories: normal breast tissue, intraductal papilloma, high-grade ductal carcinoma in situ (DCIS), invasive ductal carcinoma (IDC), and invasive lobular carcinoma (ILC). Thus, each histopathological category was represented by one patient in this proof-of-concept study. The five patients constituted the biologically independent sampling units, whereas multiple Raman spectra acquired from each patient represented repeated spectral measurements rather than independent biological samples. The sample cohort encompassed the following histopathological classifications (Table 1).

For binary machine learning classification, normal breast tissue and intraductal papilloma were assigned to the Negative class, whereas high-grade DCIS, IDC, and ILC were assigned to the Positive class. Accordingly, the dataset comprised 40 Negative spectra from two patients and 60 Positive spectra from three patients. These binary labels were defined for the purposes of the present classification task and should not be interpreted as establishing a clinically validated diagnostic grouping.

A total of 25 tissue sections were examined, with five sections associated with each patient. Twenty Raman spectra were acquired per patient, yielding 100 spectra overall. Because multiple spectra originated from the same patient, the observations were hierarchically structured and could exhibit within-patient correlations. Patient identity was therefore retained as the grouping variable for subsequent leave-one-patient-out (LOPO) cross-validation, ensuring that all spectra from a held-out patient were excluded from model training during the corresponding validation fold.

Histopathological diagnosis was independently confirmed by two experienced pathologists according to World Health Organization (WHO) classification guidelines. All procedures were approved by the institutional ethics review board, and informed consent was obtained from all participants. The relevant ethics approval identifier and approval date should be reported in the final manuscript, where applicable.

### 2.3. Raman Spectroscopic Acquisition

Tissue sections of 5 μm thickness were prepared using a microtome and mounted on calcium fluoride (CaF_2_) substrates to minimize substrate-related spectral interference. All specimens were deparaffinized using a standard xylene treatment followed by graded ethanol washes prior to spectroscopic analysis.

Raman spectra were acquired using a confocal Raman microscope (XploRA PLUS, HORIBA Scientific, Kyoto, Japan) equipped with a 785 nm near-infrared diode laser excitation source. The 785 nm wavelength was selected to reduce tissue autofluorescence while providing sufficient Raman signal intensity. A 100× objective lens (numerical aperture [NA] = 0.90, Olympus, Tokyo, Japan) was employed for spectral acquisition.

Raman spectra were recorded over the fingerprint region of 500–2000 cm^−1^ at spatially distinct measurement locations within predefined regions of interest (ROIs). The spectral integration time was 10 s per accumulation, with three consecutive accumulations acquired at each measurement location to improve the signal-to-noise ratio (SNR). The nominal laser power at the sample surface was maintained at 15 mW to limit potential laser-induced tissue damage.

Multi-point measurements were performed at spatially distinct locations within each specimen to assess intra-tissue spectral heterogeneity. For each patient, 20 Raman spectra were acquired across five tissue sections, yielding a total of 100 spectra from five patients. Importantly, these multiple spectra represent repeated, spatially resolved measurements from tissue belonging to the same patient and were not treated as independent biological replicates. Accordingly, the study comprised five biologically independent patients (*n* = 5) and 100 Raman spectral measurements (N = 100). Patient identity was retained for subsequent patient-independent leave-one-patient-out (LOPO) cross-validation, as described in the machine learning analysis section.

### 2.4. Raman Spectral Preprocessing

To mitigate the fluorescence background and instrumental noise affecting Raman spectra acquired from FFPE breast tissues, a multi-step spectral preprocessing workflow was implemented in Python 3.15 using a Jupyter Notebook v3.13.16 executed in Google Colab [18,19]. The workflow comprised spectral import, wavenumber-axis alignment, Savitzky–Golay smoothing, baseline correction, non-negativity adjustment, and vector normalization.

All spectral files (.txt format) were read using pandas, with wavenumber and intensity columns automatically detected. To ensure consistency across samples, all spectra were interpolated onto a common wavenumber axis using linear interpolation (scipy.interpolate.interp1d), with the first file serving as the reference axis. This alignment ensured consistent spectral coordinates and feature dimensions for subsequent analysis.

High-frequency instrumental noise was reduced using a Savitzky–Golay filter with a 15-point window and a second-order polynomial, implemented using scipy.signal.savgol_filter with mode = ‘nearest’ [18]. The same smoothing parameters were applied consistently to all spectra.

Fluorescence-background correction was subsequently performed using the asymmetrically reweighted penalized least-squares (arPLS) algorithm, implemented using pybaselines.Baseline.arpls [19]. The baseline smoothness parameter was set to λ = 10^5^. The estimated baseline was subtracted from each smoothed spectrum to obtain the baseline-corrected Raman signal. Negative intensity values remaining after baseline subtraction were set to zero before normalization.

Following baseline correction, spectra were subjected to L_2_ vector normalization across the analyzed spectral range using numpy.linalg.norm to reduce differences in overall spectral intensity and facilitate comparisons of relative band contributions. The resulting preprocessed spectra were used for subsequent Raman-band analysis, feature extraction, and machine learning classification.

### 2.5. Feature Extraction

Following preprocessing, 12 Raman-derived features, comprising eight characteristic band intensities and four intensity ratios, were extracted to characterize biochemical variations across breast tissue specimens [13,20]. The selected Raman bands were located at approximately 890 cm^−1^ (carbohydrates), 1003 cm^−1^ (phenylalanine), 1063 cm^−1^ (lipids), 1300 cm^−1^ (lipids), 1420 cm^−1^ (potentially overlapping nucleic-acid, protein, and lipid contributions), 1445 cm^−1^ (lipids/proteins), 1618 cm^−1^ (aromatic C=C), and 1650 cm^−1^ (Amide I). Band intensities were calculated by averaging 11 consecutive spectral data points centered on the nearest measured wavenumber to each selected band.

Four intensity ratios were subsequently calculated: I_1003_/I_1445_ (phenylalanine-to-lipid/protein-associated contributions), I_1618_/I_1063_ (aromatic C=C-to-lipid-associated contributions), I_890_/I_1063_ (carbohydrate-to-lipid-associated contributions), and I_1420_/I_1445_ (relative contributions of the corresponding spectral regions). These ratios were considered exploratory biochemical descriptors rather than validated diagnostic biomarkers. Feature extraction was performed using NumPy and pandas.

### 2.6. Machine Learning Classification and Validation

An integrated machine learning pipeline was developed for binary classification of Raman spectra into Negative (normal breast tissue and intraductal papilloma) and Positive (DCIS, IDC, and ILC) classes. The 12 Raman-derived features described in Section 2.5 were standardized using sklearn.preprocessing.StandardScaler, followed by principal component analysis (PCA) retaining 95% of cumulative explained variance. A support vector machine (SVM) with a radial basis function (RBF) kernel was employed for classification. Model hyperparameters were selected using training data only.

To prevent patient-level data leakage, leave-one-patient-out (LOPO) cross-validation was adopted instead of the original random 70/30 split. In each of the five folds, all 20 spectra from one patient were reserved for testing, while the remaining 80 spectra were used for model training. Standardization, PCA, and model fitting were performed independently within each training fold, and the resulting transformations were applied to the held-out patient without refitting.

Predictions from all five test folds were pooled to calculate spectrum-level accuracy, precision, sensitivity, specificity, F1-score, and the confusion matrix. Although LOPO ensured patient-independent testing, the limited cohort of five patients, with one patient per histopathological category, restricts the generalizability of the classification results.

## 3. Results and Discussion

### 3.1. Raman Spectral Characteristics Across Breast Tissue Pathologies

Raman spectra were acquired from five histopathological groups of FFPE breast tissues over the fingerprint region (500–2000 cm^−1^). Following preprocessing using Savitzky–Golay smoothing, arPLS baseline correction, and vector normalization, fluorescence-related background contributions were substantially reduced, allowing the underlying biochemical Raman features to be more clearly resolved and facilitating comparisons of relative spectral characteristics among the investigated tissue specimens.

**Figure 1:**
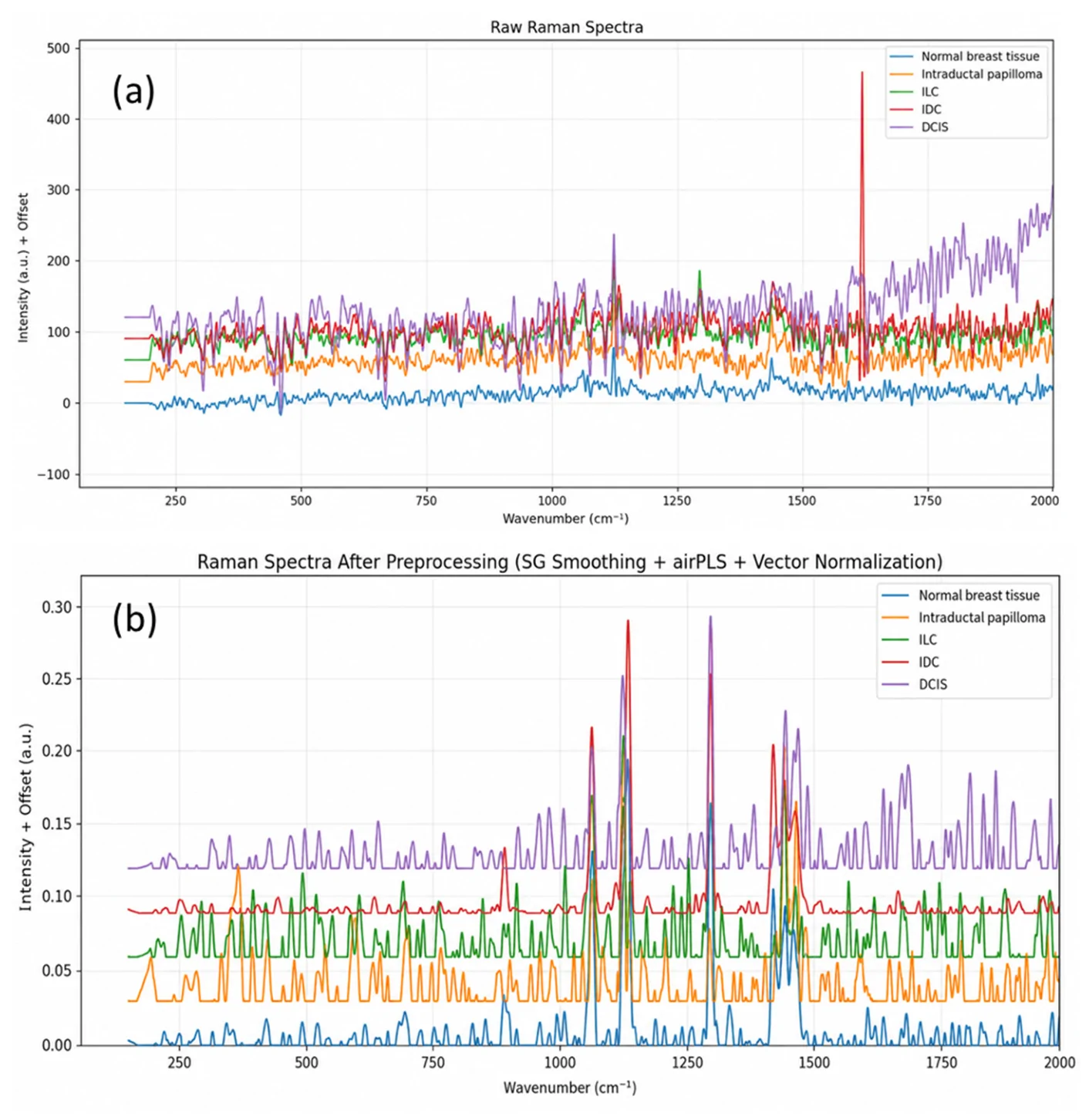
Representative Raman spectra of the five investigated breast histopathological groups. (**a**) Raw spectra showing fluorescence-related background, noise, and spectral variability before preprocessing. (**b**) Corresponding spectra after Savitzky–Golay smoothing, arPLS baseline correction, and vector normalization, revealing characteristic biochemical Raman features.

As shown in Figure 1a, the raw spectra exhibited pronounced background variation, noise, and several high-intensity spectral fluctuations that partially obscured biochemically informative Raman features. Following preprocessing, these contributions were markedly reduced, and the characteristic biochemical bands became more clearly resolved (Figure 1b). Despite differences in relative band intensities among the histopathological groups, the spectra shared a common set of Raman features associated with major tissue constituents, including lipids, proteins, carbohydrates, and nucleic acids. The relative contributions of these spectral features differed among the five investigated specimens, suggesting variations in their biochemical composition.

Normal breast tissue exhibited a predominantly lipid-rich spectral phenotype, characterized by prominent bands near 1063 cm^−1^ (C–C stretching), 1300 cm^−1^ (CH_2_ twisting), and 1445 cm^−1^ (CH_2_/CH_3_ deformation). In contrast, the phenylalanine-associated band at approximately 1003 cm^−1^ was relatively weak, resulting in a low I_1003_/I_1445_ intensity ratio. These spectral characteristics are consistent with a relatively strong lipid-associated contribution in the investigated normal breast tissue specimen.

The spectra of intraductal papilloma displayed a spectral profile with relative band intensities differing from both the investigated normal tissue and carcinoma specimens. A modest increase in the phenylalanine-associated band near 1003 cm^−1^ was observed relative to normal tissue, accompanied by moderate attenuation of lipid-associated bands. The I_1003_/I_1445_ ratio ranged from approximately 0.2 to 0.5, suggesting a higher relative contribution from phenylalanine-associated vibrations compared with the investigated normal specimen, while lipid-associated features remained evident.

High-grade DCIS exhibited additional differences in relative Raman-band intensities. Increased contributions were observed in the carbohydrate-associated region near 890 cm^−1^ and the phenylalanine-associated band near 1003 cm^−1^, accompanied by attenuation of lipid-associated bands near 1063 and 1300 cm^−1^. A feature near 1420 cm^−1^, potentially reflecting overlapping contributions from several biochemical constituents, also became more prominent. The I_1003_/I_1445_ ratio increased to approximately 0.6–1.0, indicating a higher phenylalanine-to-lipid/protein-associated spectral intensity ratio in the investigated DCIS specimen.

IDC exhibited a markedly altered Raman phenotype relative to the investigated non-invasive specimens. A prominent band near 1618 cm^−1^, assigned to aromatic C=C-associated vibrations, was observed together with an enhanced phenylalanine-associated signal near 1003 cm^−1^. Concurrently, the major lipid-associated bands near 1063 and 1300 cm^−1^ were substantially attenuated. The I_1003_/I_1445_ ratio exceeded 1.5 in the investigated IDC spectra, reflecting a pronounced difference in relative Raman-band contributions compared with the normal and benign specimens. Although the 1618 cm^−1^ feature may be informative for distinguishing the examined IDC specimen, its subtype specificity cannot be established from a single patient.

ILC exhibited a spectral profile characterized by variation in relative band intensities across the measured locations. Some locations retained appreciable lipid-associated signals, whereas others exhibited reduced lipid-associated contributions together with a relatively enhanced feature near 890 cm^−1^. These observations suggest spatial variation in the Raman spectral characteristics of the investigated ILC specimen. However, the available data do not establish that ILC tissues generally exhibit greater biochemical heterogeneity than other breast histopathological categories. Furthermore, the feature near 890 cm^−1^ cannot independently establish alterations in carbohydrate metabolism without complementary biochemical measurements.

Taken together, the spectra in Figure 1 illustrate differences in relative lipid-, protein-, nucleic-acid-, and carbohydrate-associated Raman contributions among the five representative breast histopathological specimens. A tendency toward reduced relative lipid-associated signals and enhanced contributions from selected protein- and other biomolecule-associated bands was observed across the investigated samples. However, because each histopathological category was represented by only one patient, these findings should be interpreted as exploratory specimen-associated spectral differences rather than a validated biochemical trajectory of breast cancer progression. The quantitative variation in the selected spectral features and their intensity ratios is examined in the following section.

### 3.2. Quantitative Raman Spectral Descriptors

Differences in the relative contributions of phenylalanine- and lipid/protein-associated Raman signals were observed among the investigated breast tissue specimens. In particular, the intensity ratio I_1003_/I_1445_, representing the relative contribution of the phenylalanine-associated band near 1003 cm^−1^ to the lipid/protein-associated band near 1445 cm^−1^, varied across the five histopathological categories. Table 2 summarizes the approximate ratio ranges observed in the investigated specimens.

**Figure 2:**
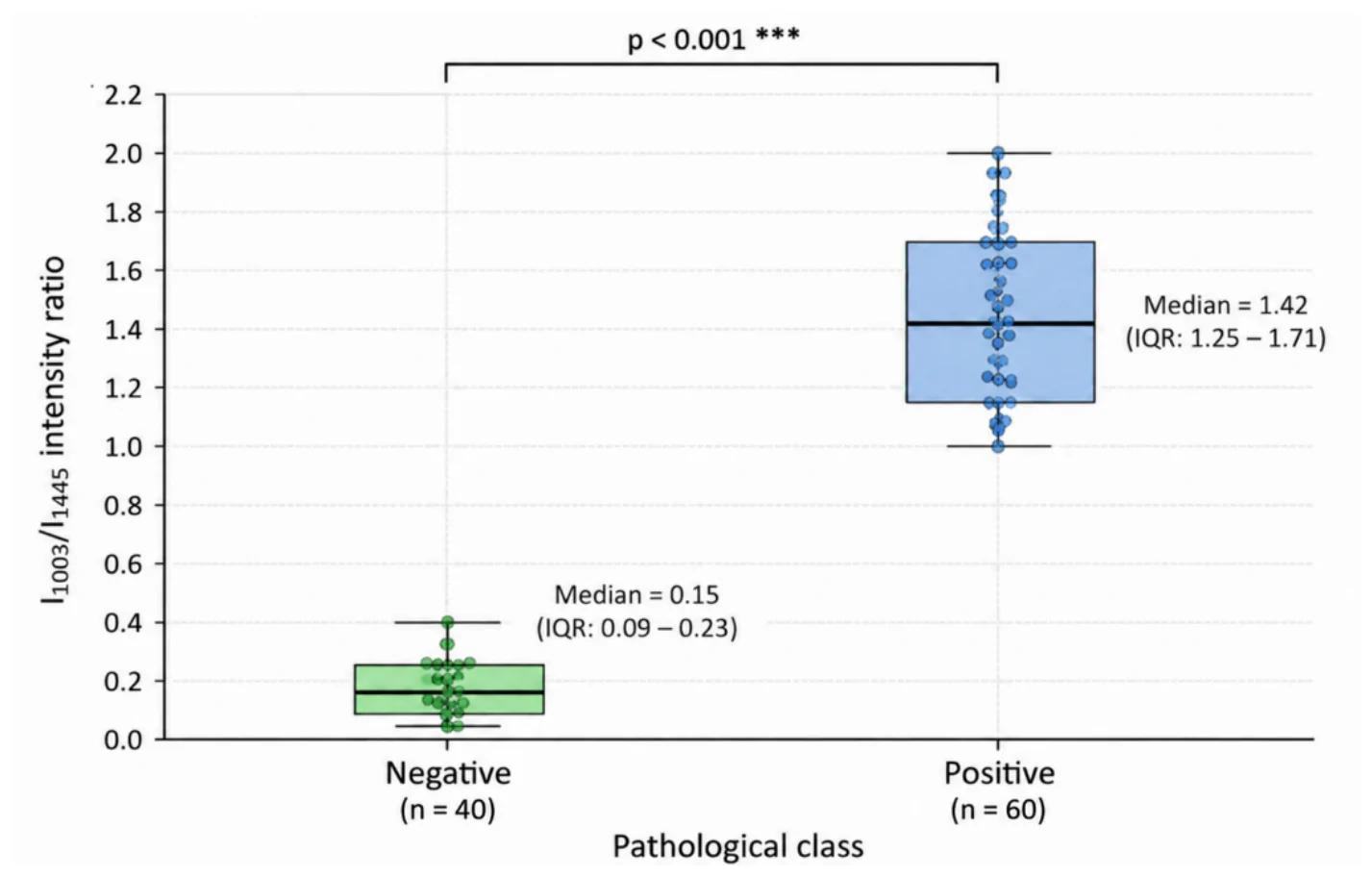
Spectrum-level distribution of the I_1003_/I_1445_ Raman intensity ratio between the Negative and Positive groups in the previously analyzed 100-spectrum subset. Each point represents an individual Raman spectrum. *** denotes a statistically significant difference at *p* < 0.001.

Figure 2 shows the distribution of the I_1003_/I_1445_ intensity ratio between the Negative and Positive spectral classes in the previously analyzed subset of 100 Raman spectra. The Negative group, comprising normal breast tissue and intraductal papilloma, exhibited a relatively narrow distribution centered at low ratio values, with a median of 0.15 (IQR: 0.09–0.23; *n* = 40 spectra). In contrast, the Positive group, comprising high-grade DCIS, IDC, and ILC, showed a higher ratio, with a median of 1.42 (IQR: 1.25–1.71; *n* = 60 spectra). The previously reported Mann–Whitney U test yielded *p* < 0.001 at the spectrum level; however, this result does not account for the clustering of spectra within patients and therefore cannot be interpreted as patient-level statistical significance.

Table 2 shows a 4-fold difference in median I_1003_/I_1445_ between the Positive and Negative spectral groups indicates substantial separation of the measured spectral distributions in this subset. This difference is consistent with the spectral observations in Figure 1, where relatively reduced lipid-associated contributions were accompanied by an enhanced phenylalanine-associated feature near 1003 cm^−1^. Nevertheless, because the 1445 cm^−1^ band contains contributions from both lipids and proteins, the ratio should be interpreted as a relative spectral descriptor rather than a direct measurement of tissue protein-to-lipid concentration.

The Positive group exhibited a broader distribution than the Negative group, as reflected by its larger interquartile range (1.25–1.71 versus 0.09–0.23). This observation may reflect differences among the three investigated Positive specimens, spatial spectral variability within specimens, or both. The present dataset does not allow these sources of variation to be reliably separated.

The two spectral distributions showed limited overlap in the analyzed subset, suggesting that I_1003_/I_1445_ contains potentially useful discriminatory information for the investigated specimens. However, the apparent separation does not establish a clinically applicable diagnostic cut-off or demonstrate generalization to independent patients. Such claims would require evaluation in a substantially larger cohort containing multiple patients per histopathological category.

Taken together, these findings identify I_1003_/I_1445_ as a potentially informative and interpretable Raman-derived descriptor of differences in relative biochemical spectral contributions among the examined breast tissue specimens. When considered alongside other Raman features, including the aromatic C=C-associated band near 1618 cm^−1^ observed in the investigated IDC specimen and the carbohydrate-associated feature near 890 cm^−1^ in the investigated ILC specimen, this ratio provides a rationale for subsequent multivariate analysis and exploratory machine learning classification.

### 3.3. Multivariate Spectral Organization and Machine Learning Classification

Principal component analysis (PCA) was applied to the standardized feature matrix comprising eight Raman peak intensities and four derived intensity ratios. In the original exploratory PCA, the first three principal components explained 72.22% of the total variance, with PC1, PC2, and PC3 accounting for 42.33%, 17.49%, and 12.40%, respectively.

**Figure 3:**
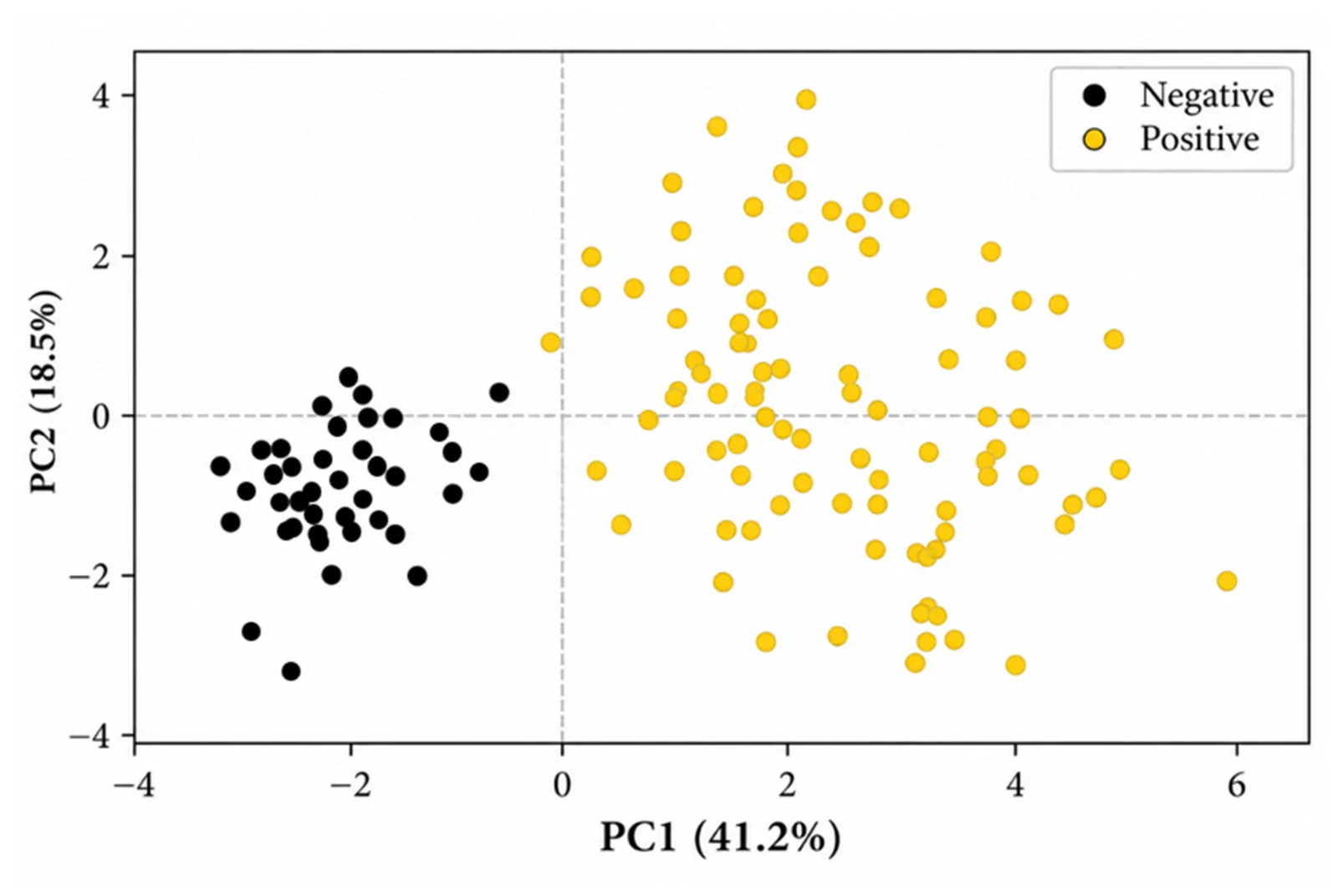
PCA score plot from the original exploratory analysis of 100 Raman spectra. PC1 and PC2 explain 42.2% and 18.5% of the total variance, respectively. The plot demonstrates substantial separation between the two spectral classes, predominantly along PC1.

As shown in Figure 3, PC1 and PC2 together accounted for 60.7% of the total variance. The 17 Negative spectra were distributed predominantly at negative PC1 scores, whereas the 60 Positive spectra were located mainly at positive PC1 scores. Although this pattern suggests differences in the multivariate Raman characteristics of the examined specimens, the separation should be interpreted cautiously because the spectra were clustered within only five patients. The broader distribution of the Positive spectra may reflect both between-patient differences and within-specimen spectral variability, which cannot be reliably distinguished in the present dataset.

To evaluate classification performance without patient-level data leakage, the original random 70/30 training–test evaluation was replaced by five-fold leave-one-patient-out (LOPO) cross-validation. An RBF-kernel support vector machine (SVM) was evaluated by holding out all 20 spectra from one patient in each fold. Across the five held-out patients, 89 of 100 spectra were correctly classified, corresponding to an overall spectrum-level accuracy of 89.0%.

The pooled LOPO predictions yielded a sensitivity of 90.0%, specificity of 87.5%, precision of 91.5%, F1-score of 90.8%, and balanced accuracy of 88.8%, as shown in Table 3. The corresponding confusion matrix comprised 35 true negatives, 5 false positives, 6 false negatives, and 54 true positives. Patient-specific classification accuracies ranged from 85% to 95%, indicating variation in performance across the five held-out specimens.

These findings suggest that the selected Raman-derived features contain discriminatory information in the investigated dataset. However, because each histopathological category was represented by a single patient, the LOPO results remain exploratory and cannot establish generalizable diagnostic performance or subtype-specific discrimination.

**Figure 4:**
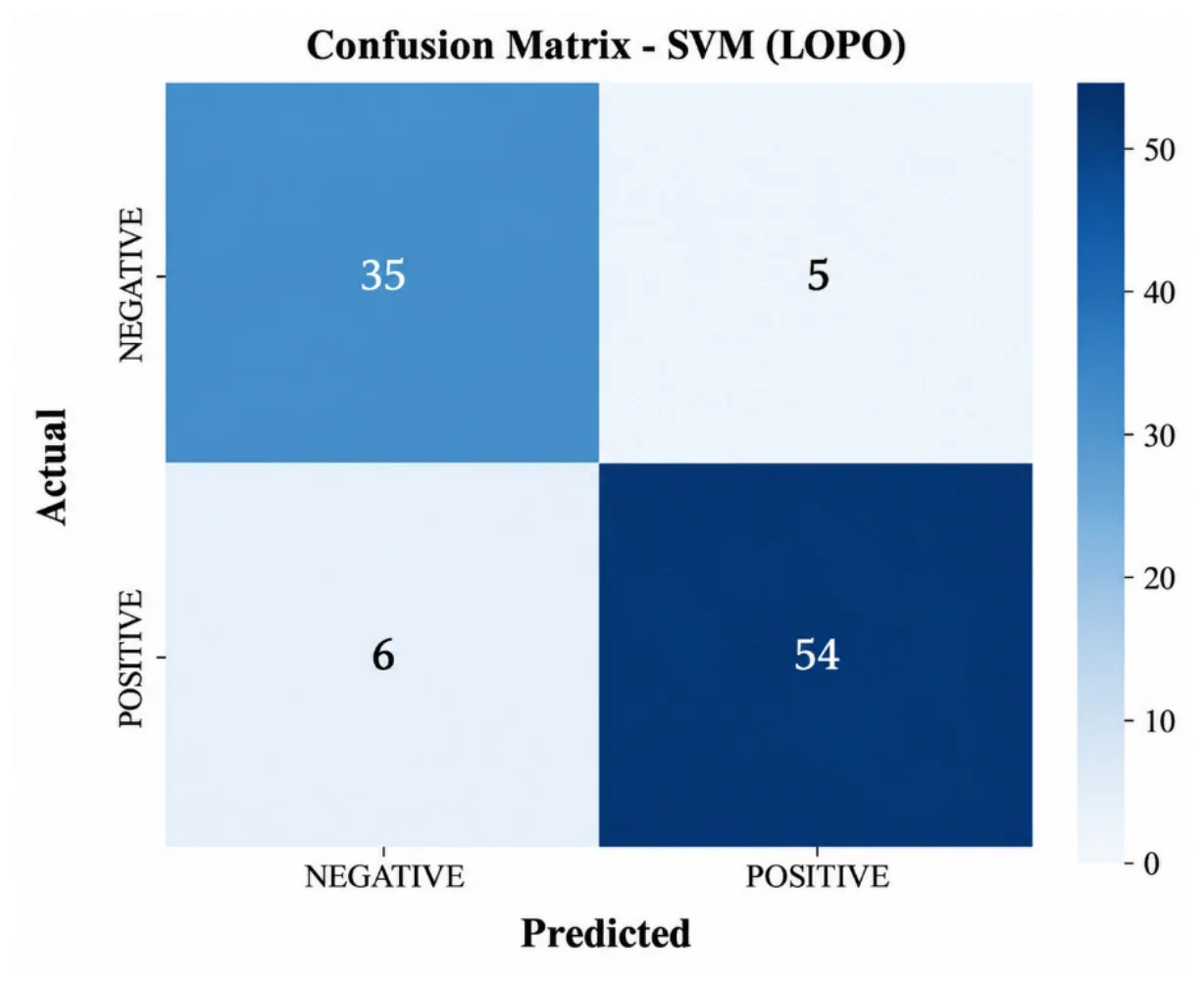
Confusion matrix of the RBF-SVM classifier evaluated using leave-one-patient-out (LOPO) cross-validation.

As shown in Figure 4, the LOPO confusion matrix revealed that the RBF-SVM classifier correctly identified 54 of 60 Positive spectra and 35 of 40 Negative spectra, corresponding to a sensitivity of 90.0% and a specificity of 87.5%, respectively. The six false-negative and five false-positive predictions indicate that some spectral characteristics overlapped between the two classes. Importantly, all spectra from each test patient were excluded from model training, reducing the risk of patient-level data leakage associated with random spectrum-level splitting. Nevertheless, the reported performance represents spectrum-level classification across five patients rather than independently validated clinical diagnostic accuracy. The limited number of patients and the representation of each histopathological category by a single individual warrant further validation in larger cohorts.

### 3.4. Discussion

The Raman spectral analysis of the investigated breast tissue pathologies revealed differences in the relative contributions of major biomolecular components among the five specimens. Attenuation of lipid-associated Raman bands near 1063, 1300, and 1445 cm^−1^ was observed in the investigated malignant specimens relative to the normal and benign specimens [5]. These spectral differences may be associated with changes in tissue cellularity and the relative contributions of lipid-rich and epithelial components [3,5]. Altered lipid metabolism is also a well-recognized feature of cancer progression, involving changes in fatty-acid synthesis, utilization, and β-oxidation pathways that support cellular proliferation [21]. Nevertheless, the present Raman measurements reflect relative tissue biochemical composition and cannot directly establish alterations in specific lipid metabolic pathways.

Concurrently, protein-associated Raman contributions near 1003 cm^−1^ and 1618–1650 cm^−1^ became more prominent in selected malignant specimens. The phenylalanine ring-breathing mode near 1003 cm^−1^ provides an informative protein-associated spectral feature [13]. The I_1003_/I_1445_ intensity ratio showed substantial differences between the investigated Negative and Positive spectral classes, suggesting its potential utility as an interpretable spectroscopic descriptor. However, because the 1445 cm^−1^ band contains both lipid- and protein-associated contributions, this ratio should not be interpreted as a direct quantitative measure of protein-to-lipid concentration or as a validated indicator of pathological progression.

Preliminary subtype-associated spectral differences were observed between the investigated IDC and ILC specimens. IDC exhibited a prominent band near 1618 cm^−1^, attributed to aromatic C=C-associated vibrations, potentially reflecting differences in protein-associated biochemical composition [5,13]. In contrast, ILC exhibited a relatively enhanced carbohydrate-associated feature near 890 cm^−1^ and variation in relative band intensities across sampling locations. The I_890_/I_1063_ ratio was previously estimated to be approximately 1.24 in ILC and 0.56 in IDC, suggesting different relative contributions from the corresponding spectral regions in these two specimens. Although altered carbohydrate metabolism and enhanced glycolytic activity are recognized characteristics of many cancers [18,20], these Raman observations do not directly demonstrate the Warburg effect or other specific metabolic mechanisms. Furthermore, because IDC and ILC were each represented by a single patient, the observed differences cannot be regarded as validated subtype-specific spectral signatures.

The revised machine learning analysis employed patient-independent leave-one-patient-out (LOPO) cross-validation to address the risk of data leakage associated with the original random spectrum-level training–test split. The RBF-SVM classifier correctly classified 89 of 100 Raman spectra, achieving an overall spectrum-level accuracy of 89.0%, sensitivity of 90.0%, specificity of 87.5%, and F1-score of 90.8%. The corresponding confusion matrix contained six false-negative and five false-positive predictions. These findings indicate that the selected Raman-derived features contained discriminatory information within the investigated dataset. However, LOPO performance should not be interpreted as independently validated clinical diagnostic accuracy, particularly given the limited number of patients and the absence of multiple independent patients within each histopathological category.

The observed classification performance is within the broad range of accuracies reported in previous Raman-based breast cancer studies [19,22,23,24]. However, direct comparisons should be made cautiously because classification performance depends strongly on sample type, cohort size, preprocessing methods, class definitions, and validation protocols. In particular, results obtained using random spectrum-level splitting are not directly comparable with patient-grouped validation. An important aspect of the present study is the integration of interpretable Raman-derived features with multivariate analysis and machine learning, allowing exploratory classification results to be discussed in relation to measurable spectral characteristics rather than relying exclusively on algorithmic predictions.

Several limitations should be acknowledged. Although 100 Raman spectra were analyzed, they originated from only five patients, with one patient representing each histopathological category. Multiple spectra from the same patient constitute spatially resolved measurements rather than independent biological replicates. Moreover, because histopathological category and patient identity were completely confounded, the present study cannot distinguish reproducible disease-specific biochemical characteristics from patient-specific variation. Although LOPO cross-validation reduced patient-level data leakage, the resulting performance estimates remain exploratory and are insufficient to establish population-level generalizability or clinical utility. The observed spectral differences and candidate biochemical descriptors therefore require confirmation in larger, independently sampled cohorts.

Future studies should prioritize larger patient cohorts, multiple independent patients per histopathological category, patient-grouped validation, multicenter evaluation, and standardized Raman acquisition and preprocessing protocols. Complementary Fourier-transform infrared spectroscopy (FTIR), histochemical analyses, and molecular or metabolomic measurements could provide additional evidence for the biochemical interpretation of the observed spectral changes. With sufficiently large and diverse datasets, explainable machine learning approaches may further support the identification and validation of robust Raman-based classification features.

Overall, this exploratory study demonstrates the feasibility of combining Raman-derived biochemical descriptors with PCA and patient-grouped SVM classification for characterizing the investigated breast tissue specimens. Although the observed spectral differences and LOPO classification results are encouraging, larger independent patient cohorts are essential before subtype-specific signatures, diagnostic thresholds, or clinical applicability can be established.

## 4. Conclusions

This study demonstrates the feasibility of combining near-infrared Raman spectroscopy with interpretable spectral analysis and machine learning for exploratory, label-free characterization and binary classification of human breast tissue specimens. A computational workflow incorporating spectral preprocessing, feature extraction, dimensionality reduction, and supervised classification was implemented and evaluated using patient-grouped cross-validation. The Raman analysis revealed differences in relative biochemical spectral contributions among the five investigated histopathological specimens, characterized by attenuation of lipid-associated bands and enhanced contributions from selected protein-associated features in the examined malignant tissues. In particular, the I_1003_/I_1445_ intensity ratio emerged as a potentially informative spectroscopic descriptor for distinguishing the investigated Negative and Positive spectral classes. Preliminary differences were also observed between the IDC and ILC specimens, particularly near 1618 cm^−1^ and 890 cm^−1^, respectively. However, these observations require confirmation across independent patients before they can be considered subtype-specific Raman signatures. Using leave-one-patient-out (LOPO) cross-validation, the RBF-SVM classifier achieved a spectrum-level accuracy of 89.0%, sensitivity of 90.0%, specificity of 87.5%, and F1-score of 90.8%. These findings suggest that the selected Raman-derived features contain discriminatory information within the investigated dataset while reducing the risk of patient-level data leakage associated with random spectrum-level splitting. Nevertheless, the 100 Raman spectra originated from only five patients, with one patient representing each histopathological category. Consequently, the observed biochemical differences and classification performance should be interpreted as exploratory proof-of-concept findings rather than clinically validated diagnostic evidence. Future studies involving larger cohorts, multiple independent patients per histopathological category, and external patient-level validation are essential to establish the robustness and generalizability of this Raman-based analytical framework.

## Figures and Tables

**Table 1:** Histopathological classification and Raman spectral distribution of patient specimens.

Patient	Histopathological Category	Binary Class	No. of Tissue Sections	No. of Raman Spectra
P1	Normal breast tissue	Negative	5	20
P2	Intraductal papilloma	Negative	5	20
P3	High-grade DCIS	Positive	5	20
P4	IDC	Positive	5	20
P5	ILC	Positive	5	20
Total	5 histopathological categories	2 Negative/3 Positive patients	25	100

**Table 2:** Observed I_1003_/I_1445_ intensity-ratio ranges across the investigated breast histopathological categories.

Pathological Group	*I*_1003_/*I*_1445_ Ratio	Disease Stage
Normal breast tissue	≤0.2	Healthy
Intraductal papilloma	0.2–0.5	Benign
High-grade DCIS	0.6–1.0	Non-invasive malignant lesion
Invasive carcinoma	≥1.5	Invasive malignant lesion

**Table 3:** Spectrum-level classification performance of the RBF-SVM model using leave-one-patient-out cross-validation.

Performance Metric	LOPO Result
Accuracy	89.0%
Sensitivity (Recall)	90.0%
Specificity	87.5%
Precision	91.5%
F1-score	90.8%
Balanced accuracy	88.8%
Correctly classified spectra	89/100

## Data Availability

The data supporting the findings of this study are available from the corresponding author upon reasonable request, subject to applicable ethical and privacy restrictions.
